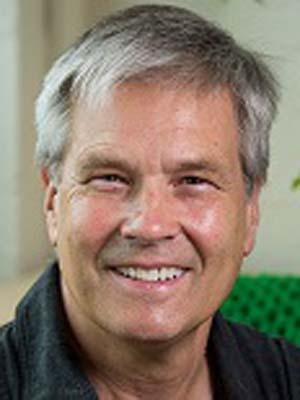# Preface for the Interfacial Transport Phenomena Collection dedicated to Professor Paul Steen

**DOI:** 10.1038/s41526-021-00170-8

**Published:** 2021-10-26

**Authors:** Ranga Narayanan, Brian Motil

**Affiliations:** 1 Department of Chemical Engineering, University of Florida, Gainesville, FL USA; 2 NASA Glenn Research Center, Cleveland, OH USA

The editorial board of *npj Microgravity* dedicates this Collection to the memory of Professor Paul Steen, Maxwell M. Upson Professor of Chemical Engineering at Cornell University. Professor Steen was an Associate Editor of this journal for over five years, providing both scholarly oversight as well as professional advice from his experience as a world-class researcher and former associate editor for the Journal of Fluid Mechanics.

Paul Steen graduated from John Hopkins University in 1981 and after a brief stint as a post-doctoral fellow at Stanford, joined Cornell in 1982 where he remained until his untimely passing on September 4, 2020. Paul’s contributions to the field of nonlinear fluid mechanics, hydrodynamic stability and interfacial fluid mechanics ranged from convective flows in porous media to stability of drops, liquid bridges and resonance patterns. He was particularly interested in conducting fluids research in the microgravity environment and did his research with the rare combination of mathematical acumen and great practical sense. Paul Steen was more than a researcher who published well and published wide. He guided many students who excelled in their own professions, owing much gratitude to Paul’s mentoring style. A devoted husband to Kyra Stepanoff and father to two daughters, Ana and Frances, Paul was also a loyal friend to many in the scientific community who cherish the time during which they knew him. This Collection is a token of the admiration that the community had for Paul and is presented as an honor to this wonderful person whose life was marked by dedication with dignity to duty.